# Laparoscopic right hemicolectomy for adult colocolic intussusception secondary to ascending colon lipoma: a case report

**DOI:** 10.1093/jscr/rjaf963

**Published:** 2025-12-06

**Authors:** Aashutosh Jha, Sujan Shrestha, Sandesh Doranga, Sushil Mishra, Binod Bade Shrestha

**Affiliations:** Department of Surgery, Manipal College of Medical Sciences, Fulbari-11, Pokhara 33700, Nepal; Department of Surgery, Manipal College of Medical Sciences, Fulbari-11, Pokhara 33700, Nepal; Department of Surgery, Manipal College of Medical Sciences, Fulbari-11, Pokhara 33700, Nepal; Department of Surgery, Manipal College of Medical Sciences, Fulbari-11, Pokhara 33700, Nepal; Department of Surgery, Manipal College of Medical Sciences, Fulbari-11, Pokhara 33700, Nepal

**Keywords:** adult intussusception, colocolic intussusception, submucosal lipoma, intestinal obstruction, case report

## Abstract

Intussusception is a rare cause of bowel obstruction in adults. Colocolic intussusception in adults is most often associated with an underlying pathological lead point. Therefore, operative intervention remains the standard of care. A 65-year-old female with three days history of abdominal pain, distention, and vomiting was found on contrast enhanced computed tomography to have colocolic intussusception with subacute small bowel obstruction. She underwent laparoscopic standard right hemicolectomy, which revealed an ascending colon lipoma as the lead point. Adult intussusception is an uncommon cause of bowel obstruction and should be managed with surgical intervention once diagnosed. Primary resection without prior reduction is the preferred in most cases due to the high likelihood of an underlying malignancy. This case report highlights the importance of surgical management. It further emphasizes the diagnostic utility of computed tomography scan in preoperative evaluation and treatment planning.

## Introduction

Intussusception refers to the invagination of a proximal bowel segment into an adjacent distal segment [1]. It is primarily a disease of childhood, most commonly occurring between 6 and 18 months of age [2] and is one of the most common causes of intestinal obstruction in this age group [3]. In contrast, only ~5% of all cases are seen in adults [1].

Patients with adult intussusception often present with signs of intestinal obstruction, and contrast enhanced computed tomography (CECT) scan of the abdomen is the investigation of choice for diagnosis and surgical planning. Since the majority of adult intussusception cases have a pathological benign or malignant lead point, surgical intervention remains the standard of care [4].

In this study, we report a rare case of adult colocolic intussusception presenting with features of intestinal obstruction, which was successfully managed by minimally invasive surgery. This case has been reported in line with the SCARE (a standardized guideline required for surgical case reports) checklist [5].

## Presentation of the case

A 65-year-old female presented to emergency department with a 3-day history of abdominal pain in the right lower quadrant along with abdominal distention and vomiting. The patient experienced relief from abdominal pain and distention after bouts of projectile vomiting. The vomiting occurred immediately after attempting to eat, although she was still able to pass stools. She had no significant past medical or surgical history. She did not consume alcohol and was a non-smoker.

On examination, the patient was alert, and her vitals were within normal limits. She was afebrile, with a pulse rate of 90 beats per minute, blood pressure of 110/70 mmHg, respiratory rate of 18 breaths per minute, and SpO_2_ of 97% on room air. On abdominal examination, her abdomen was distended, with a mildly tender, ill-defined lump about the size of an egg palpable in the right upper abdomen. All hernial sites were intact. Digital rectal examination revealed no palpable mass, and the gloved examining finger was stained with stool of normal colour. Other systemic examinations were unremarkable. The timeline of the patient’s presentation and management is summarized in Table 1.

**Table 1:** Timeline

Day	Event
Day of presentation.	65 years female presented with abdominal pain, distention and vomiting
At 1 hour of presentation	Imaging: CECT showed colocolic intussusception.
Initial management	NG decompression, IV fluids, antibiotics
At 3 hours of presentation	Emergency laparoscopic right hemicolectomy performed
Postoperative day 1–4	Uneventful recovery
Postoperative day 5	Patient discharged
2 weeks follow up	Asymptomatic
1 and 6 months follow up	Patient remained well.

**Figure 1:**
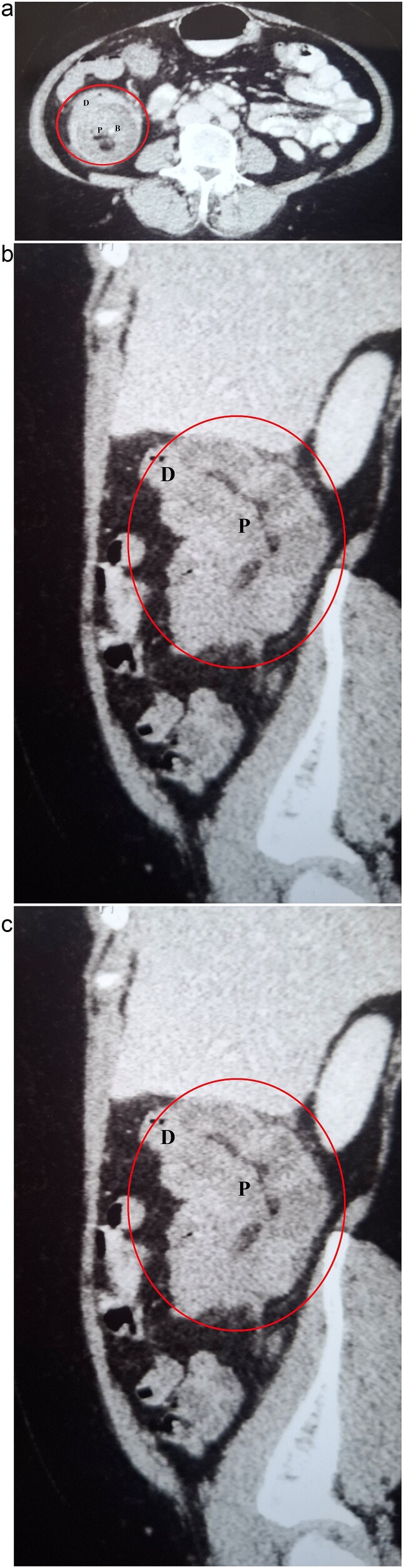
CECT abdomen demonstrating colocolic intussusception involving the ascending colon (encircled in red). (a) Axial view showing the characteristic multilayered appearance, with the proximal bowel (P) containing mesenteric blood vessels (B) and fat, surrounded by the thick-walled distal bowel (D). (b) Coronal view. (c) Sagittal view.

Her routine blood investigations were within normal limits. Ultrasound of the abdomen and pelvis showed distended bowel loops and a suspicious mass in the ascending colon. CECT scan of the abdomen was performed at 1 hour of presentation which revealed colocolic intussusception involving the ascending colon, along with dilated small bowel loops (Fig. 1). With the available clinical and radiological information, the patient was diagnosed with acute colocolic intussusception with features of subacute bowel obstruction. Following nasogastric tube decompression, she was started on intravenous fluids and prophylactic antibiotics were administered. Emergency laparoscopy, performed 3 hours after presentation, revealed a colocolic intussusception involving the ascending colon, with minimal dilated ileal loops. Minimal ascites was noted, with no visible lesions on the liver, or peritoneum, and no enlargement of mesenteric lymph nodes.

**Figure 2:**
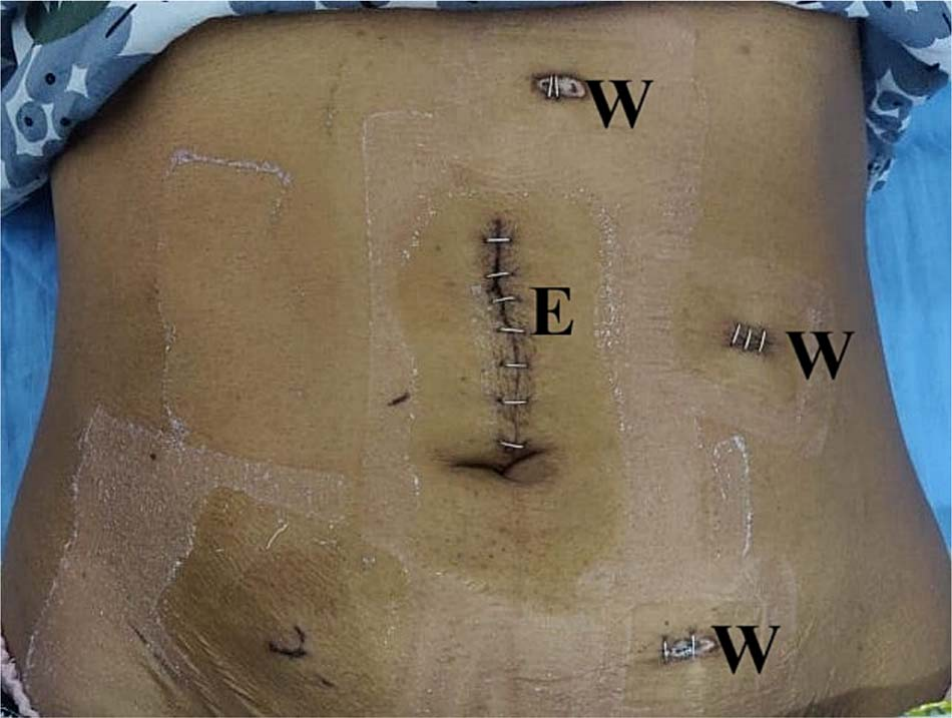
Laparoscopic port placement. E: Extraction port; W: Working port.

**Figure 3:**
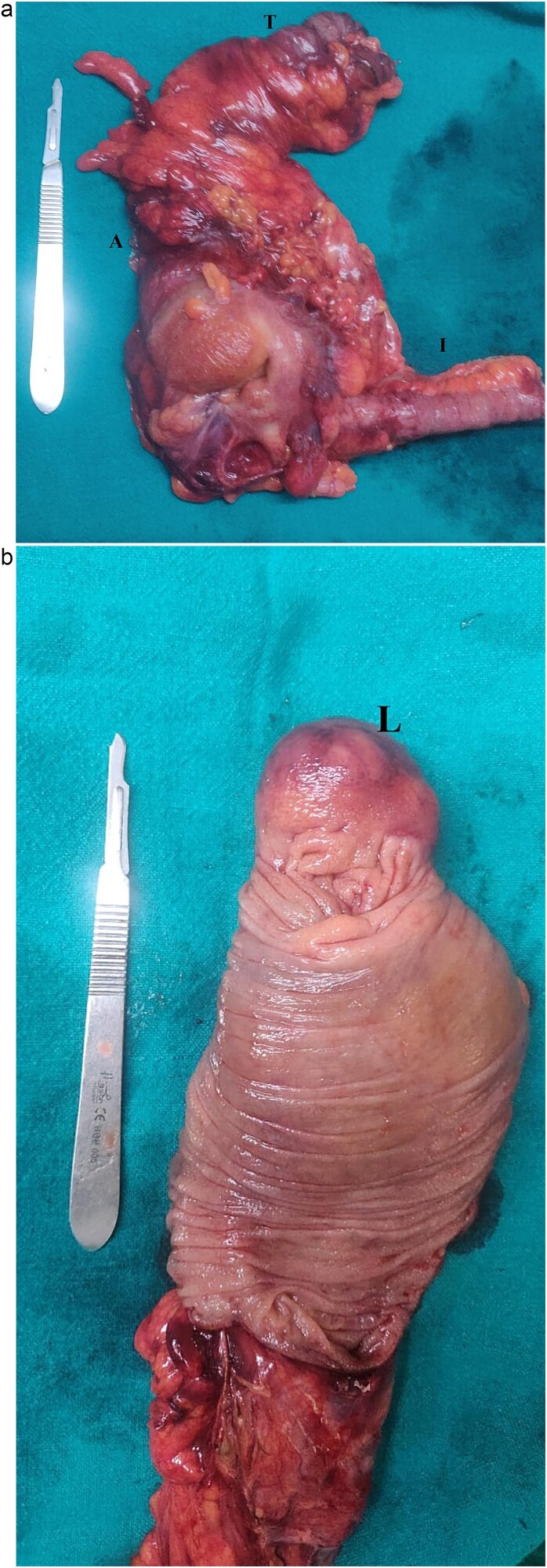
(a) Right hemicolectomy specimen showing the proximal transverse colon (T) ascending colon (A) and terminal ileum (I). (b) Cut section shows well circumscribed, sessile, nodular mass serving as the lead point (L).

**Figure 4:**
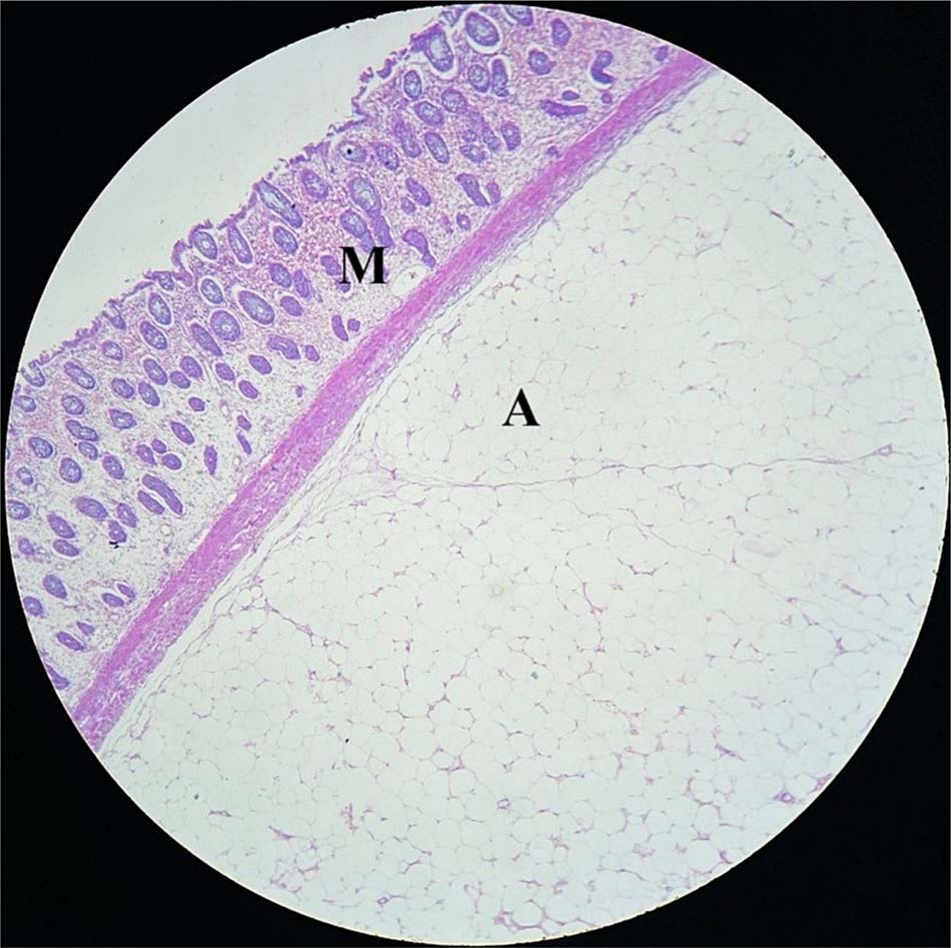
Histopathology section showing compressed and thinned colonic mucosa(M) with lobules of mature adipose tissue(A) in the submucosa separated by thick and thin fibrocollagenous septa.

Medial to lateral laparoscopic standard right hemicolectomy was performed. A 10 mm port was placed in the supraumbilical region for the camera, two 5 mm port were placed in the left iliac region and left upper quadrant, and a 10 mm port was inserted in the left lumbar region as a working port (Fig. 2). The ileocolic pedicle and the right branch of the middle colic artery were ligated flush with their respective vessels of origin, following the key concept of central vascular ligation and complete mesocolic excision. After complete dissection and mobilization of right colon, proximal transverse colon, and distal ileum, 6 cm midline incision was made by extending the previously placed supraumbilical port cephalad for specimen retrieval. An extracorporeal, hand-sewn, four layer, end-to-end ileocolic anastomosis was then performed. Gross examination of the resected specimen revealed a colocolic intussusception with 5 × 4 × 4 cm well circumscribed, sessile, light brown to pale yellow nodular mass in the ascending colon acting as the lead point (Fig. 3). Histopathological examination of the lesion showed compressed and thinned colonic mucosa with lobules of mature adipose tissue in the submucosa separated by thick and thin fibrocollagenous septa (Fig. 4).

The postoperative period was uneventful, and the patient was discharged on the 5th postoperative day. She was doing well and remained asymptomatic at her 2 weeks follow up in the outpatient department, with surgical wounds showing good healing. At her 1 month visit and again at the 6 month follow up via telephone, she continued to do well.

## Discussion

Most surgeons encounter no more than one or two cases of adult colocolic intussusception over the course of their career [6]. In adults, intussusception is typically secondary to an underlying pathologic lead point, which may be benign (lipomas, polyps, diverticula, strictures, adhesions) or malignant, most often carcinomas [7–9]. Felix *et al.* [10] estimated that 63% of adult cases are tumour related, with colonic intussusceptions commonly linked to primary malignancies and small bowel cases to benign tumours. Such lesions disrupt peristalsis, causing prolapse of the proximal bowel and mesentery into the distal lumen, leading to obstruction. If vascular flow is compromised, ischaemia, inflammation, and necrosis may ensue without timely intervention [6, 7].

Symptoms in adults are often nonspecific and commonly resemble those of chronic intestinal obstruction [11]. For instance, Lu *et al.* [12] reported a case of intussusception diagnosed only after 5 years of recurrent abdominal pain, with prior imaging failing to identify the condition. In our case, however, the patient experienced acute onset of symptoms over three days. Due to its rarity and nonspecific symptoms, diagnosis of adult intussusception is often delayed [4]. Ultrasonography is a useful tool as it is rapid, non-invasive, and reproducible [13]. Abdominal computed tomography (CT) scan is currently diagnostic modality of choice due to its high sensitivity and specificity. It can occasionally reveal the lead point [14].

Unlike pediatric cases, surgical resection is usually required in adults [4, 6, 15, 16]. En bloc resection using proper oncologic principles without prior reduction followed by anastomosis is preferred approach given the high incidence of malignancy [6, 7]. Although, reduction prior to resection might permit limited resection of the bowel, [16] it is generally discouraged [1, 6, 15–17]. Lipomas are the most common benign causes of colocolic intussusception in adults [18]. In our case, CT confirmed intussusception; however, lipoma as the lead point was not identified; a limitation that could have influenced the choice of management. We proceeded with laparoscopic standard right hemicolectomy due to high occurrence of malignancy in such cases.

Unlike the typical pediatric presentation, our case involved an adult patient with colocolic intussusception, a rare occurrence. Moreover, the condition was successfully managed with minimally invasive surgery, an approach even less frequently reported in such cases.
